# Impact of post-meal and one-time daily exercise in patient with type 2 diabetes mellitus: a randomized crossover study

**DOI:** 10.1186/s13098-017-0263-8

**Published:** 2017-08-31

**Authors:** Daizy Pahra, Nitasha Sharma, Sandhya Ghai, Abhishek Hajela, Shobhit Bhansali, Anil Bhansali

**Affiliations:** 1 National Institute of Nursing Education, Post Graduate Institute of Medical Education and Research, Chandigarh, India; 2 Department of Endocrinology, Post Graduate Institute of Medical Education and Research, Sector-12, Chandigarh, 160012 India

**Keywords:** Post-meal exercise, Glycemic control, T2DM

## Abstract

**Background:**

To evaluate the effectiveness of short-timed post-meal and one-time daily exercise on glycemic control in patients with T2DM.

**Methods:**

Sixty-four T2DM patients were randomised into crossover design. Group A (n = 32) underwent post-meal exercise (moderate-intensity brisk walking covering 1500–1600 steps for 15 min, starting 15 min after each meal) from d1 to d60 followed by one-time daily exercise (45 min pre-breakfast brisk walking at stretch covering 4500–4800 steps) from d61 to d120, while it was vice versa for the group B (n = 32). The five-point blood glucose profile was performed on d1, d30, d60, d90 and d120, and HbA1c on d1, d60 and d120. Fitness wrist band was used for step-counting to ensure the intensity of exercise and compliance to exercise protocol.

**Results:**

Group A patients showed a significant improvement in five point blood glucose profile and HbA1c after performing post-meal exercise (p < 0.001), which was mitigated after switchover to one-time daily exercise (p < 0.001). While, group B patients showed improvement in glucose profile and HbA1c (p < 0.001) after performing post-meal exercise, as compared to one-time daily exercise. Further, on pooled analysis (post-meal versus one-time daily exercise group) the beneficial effect of post-meal exercise on glucose profile and HbA1c was consistent as compared to one time daily exercise and the significance persisted on comparison between the two groups. No hypoglycemic events were noted between the groups during the study period.

**Conclusion:**

Post-meal exercise is more effective than routine one-time daily exercise for glycemic control in T2DM patients.

## Introduction

Though today’s fast paced world of urbanisation and technology has eased and reduced many of our laborious activities, it has been at the cost of rising prevalence of non-communicable diseases including obesity, metabolic syndrome, diabetes mellitus and hypertension. Type 2 diabetes mellitus (T2DM) currently accounts for majority of the world-wide burden of non-communicable diseases [1]. The incidence and prevalence of diabetes is increasing world-wide, especially in the low and middle income countries [2], with an estimate that by 2030, approximately 366 million people in the world will have diabetes [3]. In spite of plethora of anti-diabetic medications available for the treatment of T2DM, exercise and dietary modifications are, and will always remain the key advice in the management of T2DM, because it is effective at all stages of the disease, reduces HbA1c by approximately 0.51–0.89%, and is cost effective [4].

Physical activity can be in any form including aerobic exercise, resistance training or both. Current recommendations for patients with T2DM include walking or physical activity of similar intensity for at least 150 min/week, is to be performed for meaningful reduction in HbA1c [5], improvement in cardiovascular risk profile [6] and decrease in body weight [7]. However, the guidelines do not specify regarding the timing of exercise when should be undertaken during the course of the day and its relationship with fasting state versus post-meal state. Post-meal physical activity may help in greater reduction in post-prandial hyperglycemia (PPH), and may reduce the CV risk more effectivly, than overall glycemic control. The effect of post-meal exercise on postprandial glycemic excursion in T2DM was first shown by Larsen et al. They demonstrated decrease in postprandial glycemic excursions after post-breakfast exercise, however, the benefit did not persist during and following the lunch [8]. Similarly, a randomized crossover study by Nygaard et al. showed that even slow post-meal walking reduced postprandial glucose rise in healthy middle-aged women [9]. Recently, a well-designed study by Reynolds et al. showed improvement in glycemic profile after post-meal exercise, particularly in those who were consuming high carbohydrate meal [10]. However, this study was limited by a small sample size and short duration of follow-up.

The present study was aimed to evaluate the effectiveness of post-meal exercise after each meal (post-breakfast, post-lunch and post-dinner) versus one-time daily exercise on glycemic control in patients with T2DM in real-life setting with large sample size and longer duration of follow-up.

## Research design and methods

**Fig. 1:**
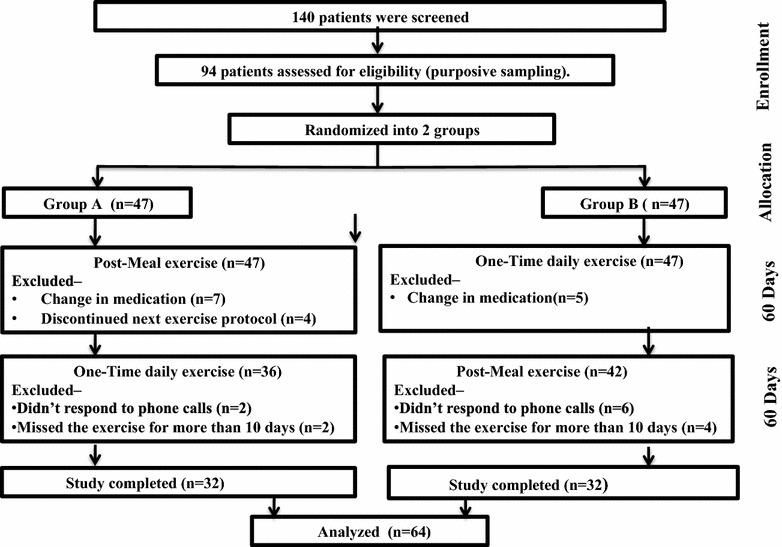
Flowchart showing study protocol

The study population included 94 adults with T2DM attending Endocrinology OPD at Post Graduate Institute of Medical Education and Research, Chandigarh from June to November 2014 and finally 64 patients were evaluable for analysis, as shown in Fig. 1. Informed consent was obtained from the study subjects, Instiutional Ethics Committee approved the study. All the patients with joint pains, fractures and those suffering from medical conditions that prevented exercise were excluded. This was a randomised crossover study design of two groups of 32 patients (group A and B), each selected by purposive sampling technique. Baseline data was collected for all patients by a structured interview including age, gender, education status, marital status, occupation, duration of diabetes, treatment history, along with height, weight (kg) and body mass index (BMI) measurement (kg/m^2^). All patients were on metformin, sulfonylurea and/or DPP-4 inhibitors along with dietary modifications. Dietary restriction recommendations were uniform during both the exercise schedule. Patients were advised to continue similar doses during the study period, unless they experienced hypoglycaemic episodes. In group A, patients performed post-meal moderate-intensity brisk walk for 15 min covering pre-defined 1500–1600 steps with each step of approximately 80 cm at a rate of 4.8 km/h after 15 min of each meal daily from day1 to d60. This was followed by one-time daily exercise, morning pre-breakfast, 45 min moderate-intensity brisk walk at a same rate covering 4500–4800 steps from d61 to d120; while in group B, it was vice versa. Fitness wrist band (Eazy Step Fitness Band, India) was used for step counting to ensure the effectiveness of the exercise program. Glycemic control was measured by self-monitoring of blood glucose (SMBG) using glucometers (Optium, Abbott, India) at 4 a.m., fasting plasma glucose (FPG), 2 h post-breakfast (PBG), 2 h post-lunch (PLG), and 2 h post-dinner glucose (PDG) on d1, d30, d60, d90, and d120. HbA1c was measured by high performance liquid chromatography (HPLC) on Bio-Rad variant II on d1, d60 and d120. Daily telephonic alert messages were sent to all patients as a reminder for the exercise schedule and to perform SMBG. We predetermined that every patient included for final analysis should have at least 90% compliance (≥108 days and >1500 or 4500 steps each day during post-meal and pre-breakfast walk, respectively) with the exercise protocol.

## Statistics

Data was analysed using SPSS (version 22.0). Non-parametric tests were used to analyse the data, as the blood glucose values were not normally distributed. All data were expressed as median and interquartile range. Baseline and post-treatment data within the groups were compared using Friedman’s test with post hoc Wilcoxon’s signed rank test (p value corrected using Bonferroni procedure). The data between the groups were analyzed using Mann–Whitney U test. The p value <0.05 was considered significant.

## Results

The schema of the study subjects is shown in Fig. 1. The socio-demographic and clinical characteristics were comparable between both the groups, with the mean age of patients in group A being 49.0 ± 9.7 years (26–74 years) and 50.7 ± 3.2 years (29–72 years) in group B, with a mean duration of diabetes 8.4 and 7.6 years, respectively. Percentage of overweight and obese subjects were comparable in both the groups (80 and 87%, p = 0.39). Before beginning the study, the self reported mean walking time was 25 min per day with 10 participants walking for 45 min or more each day. All patients were compliant to the exercise protocol as per the predetermined criteria and covered the desired steps during exercise schedule. As data shown in Table 1, patients in group A experienced significant improvement in glycemic control from d1 to d60 (post-meal exercise) followed by a significant increase in blood glucose from d61 to d120 (one-time daily exercise). On the contrary, patients in group B showed no significant improvement in glycemic control from d1 to d60 (one-time daily exercise) followed by a significant improvement in blood levels values from d61 to d120 during post-meal exercise.

**Table 1:** Blood glucose profile and HbA_1_C of the study population during the post-meal and one-time daily exercise

Group A (n = 32)								
Blood glucose (mmol/L)	Post-meal exercise				One-time daily exercise			
	Day 1	Day 30	Day 60	p	Day 60	Day 90	Day 120	p
4 a.m.	7.2 (4.7–7.6)	5.3 (4.5–5.9)	4.9 (4.4–5.4)	0.001*	4.9 (4.4–5.4)	5.9 (5.8–6.4)	6.1 (5.8–6.6)	<0.001*
FPG	7.2 (5.9–8.6)	6.5 (5.3–6.9)	5.9 (5.7–6.2)	0.001*	5.9 (5.7–6.2)	6.9 (6.6–7.4)	7.1 (6.6–7.6)	<0.001*
PBG	10.6 (8.9–12.2)	8.7 (7.5–9.9)	7.3 (6.9–8.0)	<0.001*	7.3 (6.9–8.0)	8.2 (7.7–9.2)	9.1 (8.5–10.7)	<0.001*
PLG	11.5 (9.9–12.5)	8.7 (7.5–10.8)	7.3 (7.0–8.2)	<0.001*	7.3 (7.0–8.2)	8.7 (7.5–10.8)	9.4 (7.0–9.2)	<0.001*
PDG	10.4 (8.7–12.5)	7.9 (7.1–10.8)	7.6 (7.1–10.9)	<0.001*	7.6 (7.1–10.9)	8.7 (8.0–10.2)	9.3 (8.6–10.6)	<0.001*
HbA_1_C (%)	7.9 (6.9–9.1)	–	7.0 (6.4–7.9)	<0.001*	7.0 (6.4–7.9)	–	7.6 (6.9–8.6)	<0.001*

Group B (n = 32)								
Blood glucose (mmol/L)	One-time daily exercise				Post-meal exercise			
	Day 1	Day 30	Day 60	p	Day 60	Day 90	Day 120	p
4 a.m.	6.5 (5.6–8.4)	6.6 (5.9–7.8)	6.4 (5.6–8.9)	0.026*	6.4 (5.6–8.9)	6.2 (5.8–6.9)	5.9 (5.3–6.4)	<0.001*
FPG	6.6 (5.9–8.3)	6.9 (6.3–8.7)	7.3 (7.3–6.5)	0.002*	7.3 (7.3–6.5)	6.8 (6.3–7.6)	6.6 (5.9–7.1)	0.001*
PBG	8.9 (7.4–12.0)	8.8 (7.6–12.2)	8.7 (7.7–11.8)	0.417	8.7 (7.7–11.8)	8.5 (7.6–10.4)	7.7 (7.3–9.4)	<0.001*
PLG	10.1 (8.3–12.6)	9.7 (7.9–12.3)	10.0 (8.2–12.4)	0.315	10.0 (8.2–12.4)	8.3 (7.9–10.0)	8.3 (7.6–9.3)	<0.001*
PDG	10.4 (7.8–11.4)	10.8 (8.4–11.8)	10.9 (8.4–11.7)	0.027*	10.9 (8.4–11.7)	8.9 (8.1–10.3)	8.6 (7.7–8.9)	<0.001*
HbA_1_C (%)	7.8 (7.3–8.5)	–	7.9 (7.5–9.1)	0.235	7.9 (7.5–9.1)	–	7.5 (6.8–8.4)	<0.001*

All values are expressed as median and interquartile range

*FPG* fasting plasma glucose, *PBG* post-breakfast glucose, *PLG* post-lunch glucose, *PDG* post-dinner glucose

* Significant difference from baseline

In the same pattern, HbA1c as measured on d1, d60 and d120 also showed a trend similar to the blood glucose values in both the groups. In group A, HbA1c decreased significantly from d1 to d60 (post-meal exercise) followed by a significant increase from d61 to d120 (one-time daily exercise). Though, group B showed insignificant increase in HbA1c from d1 to d60 (one-time daily exercise), there was a significant decrease from d61 to d120 (post-meal exercise). Further, on pooled analysis (post-meal versus one-time daily exercise, n = 64 in each group), the results showed significant decrease in blood glucose levels and HbA1c after post-meal exercise and significant increase in blood glucose and HbA1c after one time daily exercise (Table 2) and the significance persisted on comparison between the two groups (Table 3). No adverse events including hypoglycaemic episodes were observed during the study period in both the groups.

**Table 2:** Pooled analysis of blood glucose profile and HbA1c between post-meal exercise group and one-time daily exercise group

Blood glucose (mmol/L)	Post-meal exercise n = 64				One-time daily exercise n = 64			
	Day 1	Day 30	Day 60	p value	Day 1	Day 30	Day 60	p value
4 a.m.	6.4 (5.6–8.3)	5.9 (4.9–6.7)	5.4 (4.9–5.9)	<0.001*	5.5 (4.8–6.6)	6.1 (5.9–7.1)	6.2 (5.7–6.6)	<0.001*
FPG	7.2 (6.0–7.1)	6.7 (6.0–7.1)	6.2 (5.8–6.9)	<0.001*	6.2 (5.8–7.4)	6.9 (6.5–7.8)	7.2 (6.6–7.6)	<0.001*
PBG	9.9 (8.2–12.1)	8.7 (7.6–9.9)	7.6 (7.1–8.7)	<0.001*	7.6 (7.1–9.7)	8.2 (7.7–8.3)	8.9 (8.1–11.1)	<0.001*
PLG	10.9 (8.7–12.7)	8.5 (7.8–10.3)	7.9 (7.3–8.7)	<0.001*	8.3 (7.3–10.3)	8.9 (8.1–10.7)	9.5 (8.4–10.9)	<0.001*
PDG	10.4 (8.7–12.4)	8.9 (7.5–10.4)	7.8 (7.3–8.9)	<0.001*	7.9 (7.2–10.6)	9.3 (8.4–11.1)	9.6 (8.5–11.2)	<0.001*
HbA_1_C (%)	7.9 (6.5–8)	–	7.0 (6.4–7.9)	<0.001*	7.6 (6.8–8.1)	–	7.7 (7.2–8.6)	<0.001*

All values are expressed as median and interquartile range

*FPG* fasting plasma glucose, *PBG* post-breakfast glucose, *PLG* Post-lunch glucose, *PDG* Post-dinner glucose

* Significant difference from baseline

**Table 3:** Change in blood glucose profile and HbA1c between the groups

Blood glucose (mmol/L)	Post-meal exercise (n = 64)	One-time daily exercise (n = 64)	p value post-meal exercise v/s one-time daily exercise
∆4 a.m.	−1.1 (−2.5 to −0.3)	0.8 (0.2 to 1.4)	<0.001*
∆FPG	−0.9 (−2.3 to −0.1)	0.8 (0.2 to 1.7)	<0.001*
∆PBG	−2.3 (−4.3 to −0.6)	1.2 (0.1 to 2.2)	<0.001*
∆PLG	−2.6 (−5.4 to −0.4)	1.1 (−0.5 to 2.3)	<0.001*
∆PDG	−2.3 (−3.5 to −0.5)	1.1 (−0.1 to 2.4)	<0.001*
∆HbA_1_C (%)	−0.7 (−1.5 to −0.4)	0.3 (0.2 to 0.6)	<0.001*

All values are expressed as median and interquartile range

*FPG* fasting plasma glucose, *PBG* post-breakfast glucose, *PLG* post-lunch glucose, *PDG* post-dinner glucose

*Significant difference between the groups

## Discussion

The present study showed that post-meal exercise of 15 min after each meal resulted in a significant decrease in five-point blood glucose profile and HbA1c, relative to one-time 45 min pre-breakfast daily exercise at stretch.

Previously, many studies have been performed to evaluate various exercise regimens for glycemic control in diabetes and their effect on cardiovascular risk factors. Balducci et al. [11] showed that a supervised, facility-based exercise training program, when added to standard treatments for T2DM yielded better results than does simple counselling of the patients. Besides the duration of exercise, type of exercise performed also matters as shown in a study on 262 sedentary men and women by Church et al. In this study, reduction in HbA1c achieved by either aerobic or resistance exercise done alone was less than that achieved when both were done together [12]. In the present study, we preferred brisk walk for the exercise protocol because it is easy to perform, inexpensive and is an aerobic activity, hence helps in better mobilisation of body fat stores [7]. Further, by walking at a speed of 4.8 km/h, our patients could qualify for moderate—intensity exercise of 3.3 MET.

Though, exercise in any form is beneficial for glycemic control, irrespective of the duration of disease, gender and age of the patients, results vary according to the protocol followed [13]. Many previous studies devoted to evaluate the effect of post-meal exercise on glycemic control in patients with diabetes were done under supervised settings. In the study by DiPietro et al. inactive elderly subjects aged ≥60 years were subjected to different exercise protocols, including 45 min morning walk, 45 min afternoon walk or post-meal exercise for 15 min over a period of 2 days inside a whole room calorimeter. The post-meal exercise protocol significantly reduced post-dinner blood glucose values over a period of 3 h as compared to 45 min walk at stretch [14]. Another study by Caron et al. also demonstrated the beneficial effect of post-breakfast exercise in type 1 diabetes patients on post-breakfast and post-lunch blood glucose values [15]. Unlike both these studies which had a supervised two-day exercise protocol, our study was performed in real-life settings and allowed all the patients to follow their daily routine activities throughout the 120-day study period.

Few crossover studies, though of short duration, have been done previously to evaluate the role of post-meal exercise on glycemic control. Aadland et al. [16] performed a crossover study on nine healthy subjects who underwent three different post-meal exercise protocols on three consecutive days after meals consisting of cornflakes. The study highlighted the beneficial effects of post-meal light intensity exercise on postprandial glucose excursions [16]. Similarly, Lunde et al. also showed the reduction in blood glucose level after post-meal exercise in each session of 20 and 40 min 1 week apart in 11 female Pakistani immigrants [17]. Further, Reynolds et al. also conducted a cross-over study with a wash-out of 30 days before switch-over to other exercise schedule and showed a significant decrease in iAUC glucose following post-meal exercise than on a single daily walk. Moreover, this was more evident after the evening meal, when predominantly carbohydrates were consumed and sedentary behaviours were highest [10]. The present study also had a crossover design with two groups consisting of 32 patients each and showed that post-meal exercise resulted in significant reduction in postprandial glycemic levels, which were attenuated after switching over to one-time daily exercise. However, patients who initiated with one time daily exercise schedule did not have significant benefit in glycemic control, because some of these participants were already performing exercise as a part of their daily routine. However, these patients after crossing over to the post-meal exercise programme had significantly better glycemic control, thereby establishing the superiority of post-meal exercise over one-time daily exercise. Though post-meal exercise could explain the improvement in postprandial glycemic excursions, reduction in fasting plasma glucose with post-meal exercise could be expounded by the ‘carry over’ effect of well-controlled post-meal plasma glucose (including post-dinner) for the entire day.

The timing of initiation of post-meal exercise is also important as starting exercise 15, 30, 45 min and beyond after meals has variable influence on the post-prandial glycemic excursions. As shown by Larsen et al. starting of exercise 30 min after meals effectively attenuates the glucose surge as compared to early or late post-prandial walks [8]. We showed even early post-meal (15 min after meal) exercise also has a beneficial effect on glucose profile.

Further, there was also a significant decrease in HbA1c during post-meal exercise protocol. This could well be explained by the work of Monnier et al. [18], which showed that contribution of postprandial glucose values towards HbA1c decreased from 70% at lower HbA1c values (<7.3%) to 30% at higher HbA1c values (>10.2%). Since, the mean HbA1c level in our study at inclusions was on the lower side (HbA1c-7.6%), thereby post-prandial glucose levels had a relatively higher contribution towards it, and henceforth the reduced post-meal glycemic excursions following post-meal exercise further improved HbA1c levels in our study subjects.

The mechanisms contributing to improved glycemic control after exercise at anytime include increased peripheral glucose consumption due to enhanced muscle glycogenolysis (contraction-mediated) as well as insulin-independent glucose uptake in the muscle by increasing GLUT 4 (glucose transporter) expression on cell surface through activation of 5′–AMP-activated protein kinase [19]. Further, with the post-meal exercise additional mechanisms include suppression of hepatic glucose output and fatty acid oxidation due to rising glucose and consequent increase in insulin-glucagon ratio following meal [20]. In addition, despite similar improvement in cardiorespiratory fitness irrespective of post-meal or one time daily walk, the glycemic profile is better with post-meal exercise than one time daily exercise. It is attributed to increased energy expenditure with multiple short daily sessions, as compared to single daily session which is associated with decreased energy expenditure [21]. Further, decrease in the splanchnic blood flow which coincides with the onset of post-meal exercise, results in slowing of absorption of nutrients from the gastrointestinal tract and consequent attenuation of post-prandial glycemic excursions. Moreover, this may also abate hypoglycemia due to slow and persistent absorption of nutrients from the gastrointestinal tract. On the contrary, during pre-meal exercise hepatic glucose output continues to remain elevated and these patients are at risk of hypoglycemia particularly when they are on sulfonylureas. The proposed mechanisms during post-meal exercise contribute to short period of ‘β-cell rest’ which may be beneficial in the long term glycemic control.

Strengths of our study include a large sample size, with complete adherence to the exercise protocol over the entire study period of 120 days, in comparison to previous studies of small sample size and short duration. Further, the double crossover design helped to mitigate the legacy effect of previous exercise schedule at the time of switchover to the next protocol. All previous studies have followed a supervised study protocol in a controlled setting, unlike the present study where the subjects followed the exercise protocol in real-life settings without their daily routine getting disturbed. We also made appropriate use of technology in form of mobile phone messages and step counting by fitness wrist band to ensure adherence towards exercise protocol. As far as the limitations are concerned, we failed to perform carbohydrate counting as a percentage of total energy, did not use continuous glucose monitoring system (CGMS) to define glycemic excursions and lack of “wash-out” period during cross-over of the study groups.

In conclusion, moderate-intensity brisk walking for 15 min after each meal is more beneficial to control blood glucose in patients with T2DM, as compared to routine 45 min walk at stretch. The compliance towards exercise can be strengthened by regular reminder messages on mobile phones and by step counting, along with counselling at each visit.

## Abbreviations

T2DM: type 2 diabetes mellitus
HbA1c: glycated hemoglobin
d: day
MET: metabolic equivalent
FPG: fasting plasma glucose
PBG: post-breakfast glucose
PLG: post lunch glucose
PDG: post dinner glucose
CGMS: continuous glucose monitoring system
